# Supplementary material for: Research on technology prospect risk of high-tech projects based on patent analysis
Source: PLoS One. 2020 Oct 5;15(10):e0240050. doi: 10.1371/journal.pone.0240050 (PMC7535056; doi:10.1371/journal.pone.0240050)
Supplement: Attachment — Submitted filename: Revison notes.docx [file pone.0240050.s002.docx]

Dear Editor,

We are very sorry for not returning revision to you sooner. Thank you for your letter of decision and for the referee’s comments concerning our manuscript entitled **“Research on technology prospect risk of high-tech projects based on patent analysis” (ID PONE-D-20-04089).** We have studied the comments carefully and have made correction which we hope meet with their approval.

We have read your emendations very carefully and [made](http://dj.iciba.com/Made/) the following corrections:

**Editor comment:** Thank you for stating the following in the Acknowledgments Section of your manuscript: "This work was supported by Chinese National Social Science Foundation funded by the Chinese government (No.15CTQ031)."

We note that you have provided funding information that is not currently declared in your Funding Statement. However, funding information should not appear in the Acknowledgments section or other areas of your manuscript. We will only publish funding information present in the Funding Statement section of the online submission form.

Please remove any funding-related text from the manuscript and let us know how you would like to update your Funding Statement. Currently, your Funding Statement reads as follows: "No"

**Response:** According to the Editor's suggestion, we have deleted the text related with the Funding- form our manuscript and updated the Funding information in our Funding Statement.

**Reviewer #1**

**Comment 1:** The paper investigates an interesting topic. I would suggest to expand the discussion or to integrate the discussion with conclusion in one section (to avoid many short sections), and to emphasize also contribution to the knowledge (in terms of extension of past studies). Author might also make a note to the usage of patent analysis in different sectors (from marketing to material science and so on).

**Response:** According to the Reviewer's suggestion, we have integrate the discussion with conclusion in one section. At the same time, we emphasize contribution to the knowledge. You can see “5. Discussion and Conclusion section”.

**Comment 2:**  I would also suggest to use image with a better resolution.

**Response:** As the reviewer’s suggestion, we revised our figures. You can see the figure 4-7.

**Reviewer #2**

**Comment 1:** The introduction is clearly written and explains well the problem and the motivation for this work. The related work has been largely addressed with a sufficient number of quotations. The methodology is explained in sufficient detail. However, the empirical study is lacking in detail, especially in providing a background of the application field (optical communication).

**Response:** According to the Reviewer's suggestion, we added background of the application in our manuscript. You can see the line 284-305.

**Comment 2:** Moreover, it is complicated to understand how the keywords were found. The search query used for patent extraction from the USPTO database is missing. The process of determination of technical keywords and acquisition of patent in empirical study, needs to be addressed.

**Response:** As the reviewer’s suggestion, we added the 4.1 Data collection section, Table 1 and Figure 3.

**Comment 3:** It would be good to describe all stages of framework application in more details during the empirical study.

**Response:** Thank you for reviewer’s suggestion. We have described all stages of the framework application in the methods section. Our purpose is that the relevant personnel can repeat our work in the future. We hope to provide an idea and method to reduce the technology prospect risk for high-tech enterprises. Therefore, we described the important steps in the empirical study section and showed the important results. This will make the structure of our manuscript more reasonable to suit the requirements of PLoS One journal.

**Comment 4:** The discussion should also focus on the results of the patent analysis carried out during the empirical study in order to provide evidence on the effectiveness of the presented framework.

**Response:** we revised this section. You can see line 355-367. The validity of the experimental results can be verified by the actual implementation of the project. Through the implementation of the "ASON Automatic Switching Optical Network Node Equipment Development and System" project, P Company has successfully developed ASON node equipment with T-bit switching capacity, which has been applied to the National High-tech Plan Project "High-performance Broadband Information Network" and many other information networks.At the same time,P Company has also launched the converged intelligent optical network solution to provide a full range of support for the evolution of operators' existing transmission networks to the next generation of intelligent optical networks.

The project has achieved innovative results in the ASON node equipment frame structure, large cross-capacity non-blocking cross-over matrix construction and so on,which have been applied for and authorized more than 20 invention patents. Moreover, P Company has also participated in the formulation and revision of a number of ASON-related international standards. In addition, P Company has not been subject to patent infringement lawsuits for any of the final results.

We are looking forward to hearing from you soon. Thank you very much and with best regards.

Yours sincerely,

Dr. Liwei Zhang and Dr. Zhihui Liu
